# Supplementary material for: Outer membrane vesicles hijack TIM-1 for cellular uptake
Source: PLoS Pathog. 2026 May 26;22(5):e1014256. doi: 10.1371/journal.ppat.1014256 (PMC13225658; doi:10.1371/journal.ppat.1014256)
Supplement: S1 Text — Table B. GO molecular function enrichment for OMV-binding receptors. Table C. OMV size. Table D. Surface validation of receptor overexpression cell lines. Table E. Bacterial strains used in the study. Table F. Cell lines used for this study. Fig A. Anti–TIM-1 antibody reduces OMV uptake across TIM-1–expressing cell lines. Fig B. Human TIM-1 (hTIM-1) interacts with phosphatidylethanolamine (PE). Fig C. LPS does not indiscriminately bind receptors. Fig D. OMVs from LPS modified E. coli strains are internalized by A549 and Caco-2 cells. Fig E. Overexpression of TIM-1 in THP-1 monocytes. (DOCX) [file ppat.1014256.s001.docx]

**S1 Text**

**Table A. OMV-binding proteins.** Twenty-six proteins with a normalized relative luminescence unit (RLU) value of >0.01 in both screening replicates.

| Protein ID | RLU (Rep 1) | RLU (Rep 2) |
| --- | --- | --- |
| WBP1 | 0.157 | 0.170 |
| APLP2 | 0.095 | 0.105 |
| TIM-1 | 0.071 | 0.049 |
| TIM4 | 0.057 | 0.046 |
| LSAMP | 0.041 | 0.060 |
| CD300LG | 0.061 | 0.025 |
| NRP1 | 0.049 | 0.036 |
| FCRL2 | 0.056 | 0.025 |
| CD207 | 0.012 | 0.055 |
| LILRA1 | 0.032 | 0.032 |
| SELL | 0.052 | 0.012 |
| CD300A | 0.043 | 0.019 |
| NRP2 | 0.031 | 0.031 |
| KIAA0319L | 0.011 | 0.044 |
| CDCP1 | 0.029 | 0.021 |
| PLXDC1 | 0.022 | 0.028 |
| TREM2 | 0.028 | 0.015 |
| MMP16 | 0.022 | 0.020 |
| CX3CL1 | 0.018 | 0.021 |
| MXRA5 | 0.020 | 0.019 |
| SIGLEC5 | 0.026 | 0.011 |
| SIGLEC6 | 0.025 | 0.011 |
| FCRL6 | 0.017 | 0.017 |
| KIRREL2 | 0.021 | 0.012 |
| CD300C | 0.013 | 0.017 |
| LOC195977 | 0.017 | 0.011 |

**Table B. GO molecular function enrichment for OMV-binding receptors.** Enriched GO terms among the 26 hits are shown with raw P values and false discovery rate (FDR), calculated against the 1,513 proteins screened.

| GO Molecular Function | raw P value | FDR |
| --- | --- | --- |
| Phosphatidylserine binding | 6.26E-06 | 3.43E-03 |
| Modified amino acid binding | 4.21E-05 | 9.21E-03 |
| Heparin binding | 4.08E-06 | 4.47E-03 |
| Glycosaminoglycan binding | 1.28E-05 | 3.50E-03 |
| Sulfur compound binding | 8.11E-06 | 2.96E-03 |

**Table C. OMV size.** Z-average diameter (d. nm) and polydispersity index (PdI) of OMVs as measured by dynamic light scattering.

| Vesicle | Z-Avg | PdI |
| --- | --- | --- |
| *E. coli* K-12 (untreated) | 92.35 | 0.258 |
| *E. coli* K-12 (PMBN) | 89.92 | 0.246 |
| *E. coli* K-12 (proteinase K) | 93.29 | 0.262 |
| *E. coli* K-12 (Mg^2+^) | 112.6 | 0.447 |
| *E. coli* K-12 (∆*waaC*) | 124.1 | 0.266 |
| *E. coli* K-12 (+*wbbL*) | 133.39 | 0.196 |
| *E. coli* K-12 (+*mcr-1*) | 90.47 | 0.278 |
| LPS (2mg/mL) | 323.2 | 0.382 |
| LPS (0.5mg/mL) | 309.4 | 0.291 |
| LPS (0.125mg/mL) | 5.306 | 0.309 |

**Table D.** Surface validation of receptor overexpression cell lines.

| Cell Line | Vector | Antibody Treatment | MFI | Population receptor positive (%) |
| --- | --- | --- | --- | --- |
| A549 | Control | Anti-NRP1 | 876 | 4.09 |
| A549 | NRP1 | Anti-NRP1 | 2451 | 36.8 |
| A549 | Control | Anti-TIM-1 | 3388 | 93.7 |
| A549 | TIM-1 | Anti-TIM-1 | 6272 | 98.8 |
| A549 | Control | Anti-TIM-4 | 3 | 0.62 |
| A549 | TIM-4 | Anti-TIM-4 | 2858 | 64.8 |
| A549 ∆TIM-1 | Control | Anti-TIM-1 | 249 | 9.11 |
| A549 ∆TIM-1 | TIM-1 | Anti-TIM-1 | 1117 | 41.1 |
| A549 ∆TIM-1 | TIM-1 (AAAA mutant) | Anti-TIM-1 | 1112 | 47.4 |
| THP-1 | Control | Anti-TIM-1 | 15 | 2.99 |
| THP-1 | TIM-1 | Anti-TIM-1 | 2946 | 84.7 |
| THP-1 | Control | Anti-TIM-4 | 29 | 0.47 |
| THP-1 | TIM-4 | Anti-TIM-4 | 2946 | 90.5 |

**Table E.** Bacterial strains used in the study.

| Bacterial species/strains | Genotype/Other Information |
| --- | --- |
| *E. coli* BW25113 | Wildtype *E. coli* |
| *E. coli* BW25113 | *E. coli* + pLMG18-ssDsbA: NanoLuc |
| *E. coli* BW25113 ∆*tolQ* | ∆t*olQ*, Keio collection, OMV overproducer |
| *E. coli* BW25113 ∆*tolQ* | ∆*tolQ* + pACYC117-*wbbL* |
| *E. coli* BW25113 ∆*waaC* | ∆waaC, Keio collection, truncated LPS |
| *E. coli* BW25113 ∆*tolQ* | ∆*tolQ* + pBAD24-*mcr-1* |
| *F. nucleatum* ATCC 23726 | Wildtype Fn23726 |
| *E. coli* (O157:H7) ATCC 35150 | Wildtype enterohemorrhagic *E. coli* (EHEC) |
| *A. baumannii* ATCC 17978 | Wildtype Ab17978 |
| *P. aeruginosa* PA14 | Wildtype PA14 |
| *E. coli* LF82 | Adherent-invasive *E. coli* (AIEC) |
| *S.* Typhimurium ATCC 14028 | Wildtype *Salmonella enterica* subsp. *enterica* serovar Typhimurium |

**Table F.** Cancer cell lines used for this study.

| Cell Line | Genotype/Other Information |
| --- | --- |
| A549 (ATCC) | Human lung epithelial cell line |
| A549 (ATCC) | A549-Vector Control-pBac |
| A549 (ATCC) | A549-TIM-1-pBac |
| A549 (ATCC) | A549-TIM-4-pBac |
| A549 (ATCC) | A549-APLP2-pBac |
| A549 (ATCC) | A549-NRP1-pBac |
| Caco-2 (ATCC) | Human colon epithelial cell line |
| Caco-2 (ATCC) | Caco-2-Vector Control-pBac |
| Caco-2 (ATCC) | Caco-2-TIM-1-pBac |
| Caco-2 (ATCC) | Caco-2-TIM-4-pBac |
| Caco-2 (ATCC) | Caco-2-APLP2-pBac |
| Caco-2 (ATCC) | Caco-2-NRP1-pBac |
| THP-1 (ATCC) | Human monocyte cell line |
| THP-1 (ATCC) | THP-1-Vector Control-pBac |
| THP-1 (ATCC) | THP-1-TIM-1-pBac |
| 769-P (ATCC) | Human kidney epithelial cell line |
| ACHN (ATCC) | Human kidney epithelial cell line |
| IGROV-1 (DCTD) | Human ovary epithelial cell line |
| Suit-2 (JCRB) | Human pancreas epithelial cell line |
| Huh-7 (JCRB) | Human liver epithelial cell line |
| SNU-449 (ATCC) | Human liver epithelial cell line |
| 786-O (ATCC) | Human kidney epithelial cell line |
| HCC1534 (UTSW) | Human lung epithelial cell line |
| A-704 (ATCC) | Human kidney epithelial cell line |
| Cal-51 (DSMZ) | Human breast epithelial cell line |
| FU97 (JCRB) | Human stomach epithelial cell line |
| HeLa (ATCC) | Human cervix epithelial cell line |
| HCT-116 (ATCC) | Human colon epithelial cell line |
| HT-29 (ATCC) | Human colon epithelial cell line |
| RPTEC (ATCC) | Human primary renal proximal tubule epithelial cells |


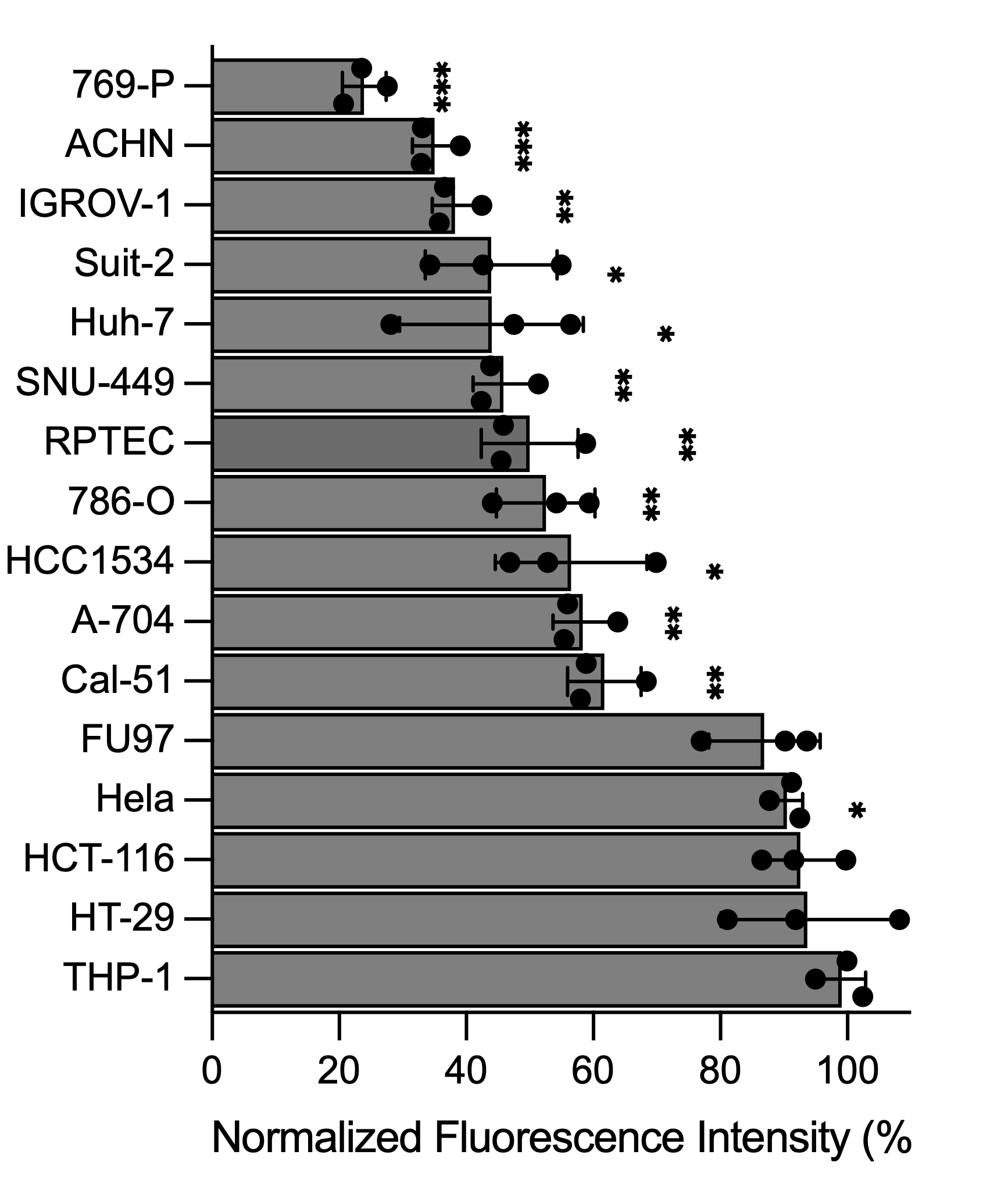


**Fig A. Anti–TIM-1 antibody reduces OMV uptake across TIM-1–expressing cell lines.** Cells were treated with anti–TIM-1 monoclonal antibody (10 µg/mL) or isotype control 30 mins before adding DiO-labeled *E. coli* K-12 OMVs. Uptake was quantified by flow cytometry after 2 hours with trypan blue quenching and normalized to isotype (= 100%). Bars show mean ± std deviation. TIM-1–positive lines (769-P, ACHN, IGROV-1, Suit-2, Huh-7, RPTEC, SNU-449, 786-O, HCC1534, A-704, Cal-51, Caco-2, A549) showed ≥40% reduction with anti–TIM-1, whereas TIM-1–negative lines (HeLa, HCT-116, HT-29, THP-1) were largely unaffected. Bars represent mean ± s.d, n = 3, *** P < 0.001, ** P < 0.01, * P < 0.05 compared to an isotype control, using one-sample t-test vs 100% (two-sided).


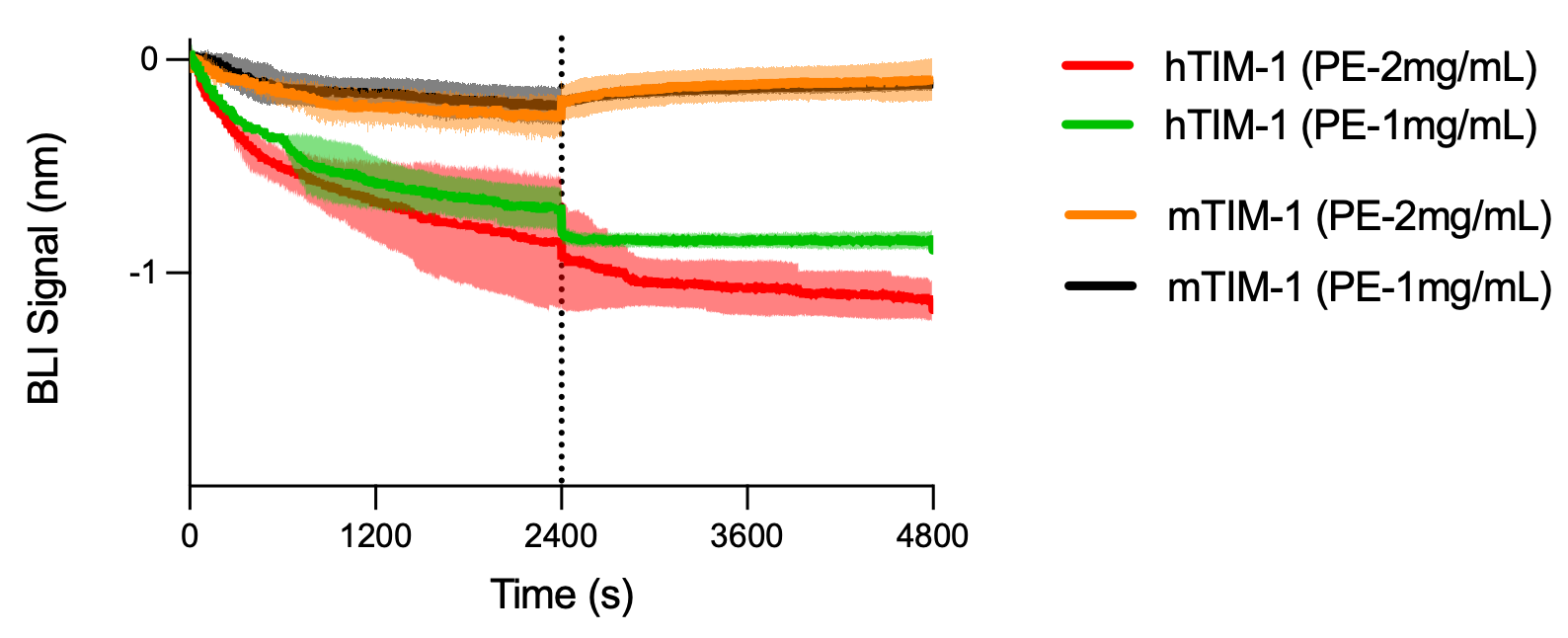


**Fig B. Human TIM-1(hTIM-1) interacts with phosphatidylethanolamine (PE).** PE binding is observed for hTIM-1 but not mouse TIM-1 (mTIM-1) as detected by BLI. Solid line represents the mean and the shaded band is ± s.d. of at least two biological replicates


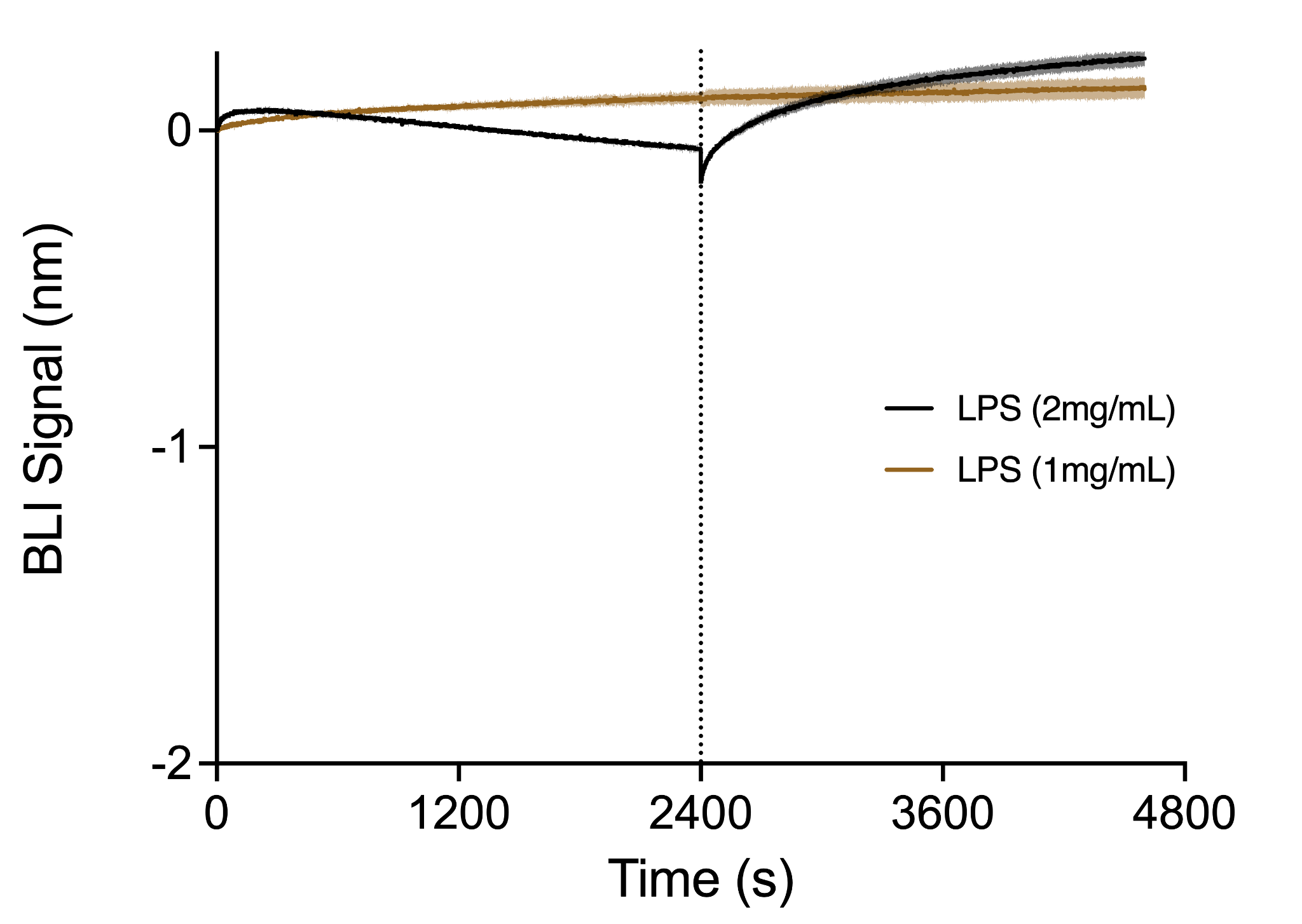


**Fig C. LPS does not indiscriminately bind receptors.** Human EGFR did not demonstrate binding to LPS at concentrations shown to interact with TIM-1. Solid lines represent the mean and the shaded band is ± s.d. of at least two replicates.

**
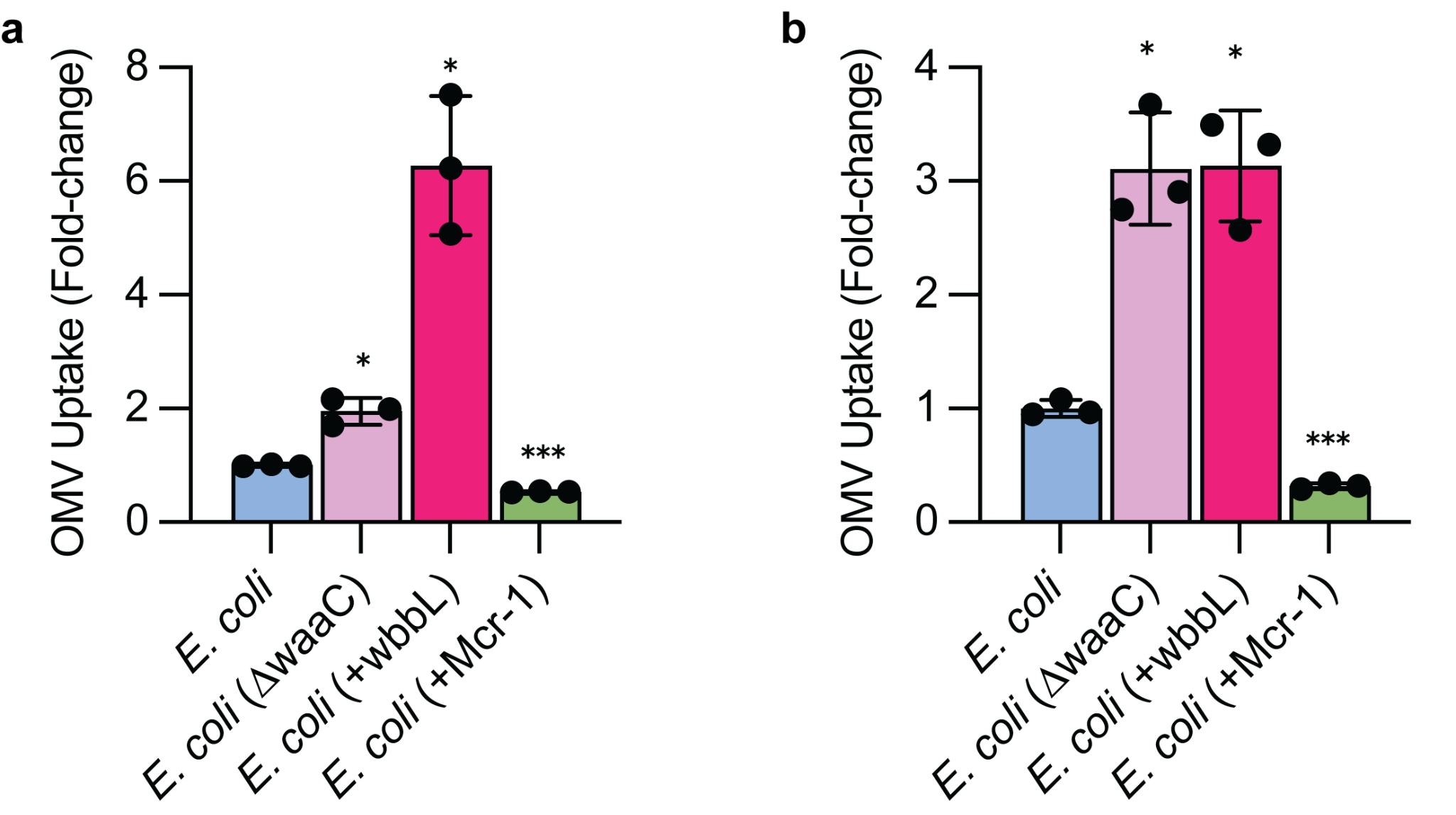
Fig D. OMVs from LPS modified *E. coli* strains are internalized by A549 and Caco-2 cells. (A)** Relative OMV uptake in A549 cells after 2 hours using the indicated OMV species. **(B)** Relative OMV uptake as in (A) except in Caco-2 cells. Values are normalized to an *E. coli* control strain with unmodified LPS structure (= 100%) and analyzed using one-sample t-test vs 100% (two-sided, * P < 0.05, *** P < 0.001). Bars show mean ± s.d. (n = 3)**.**


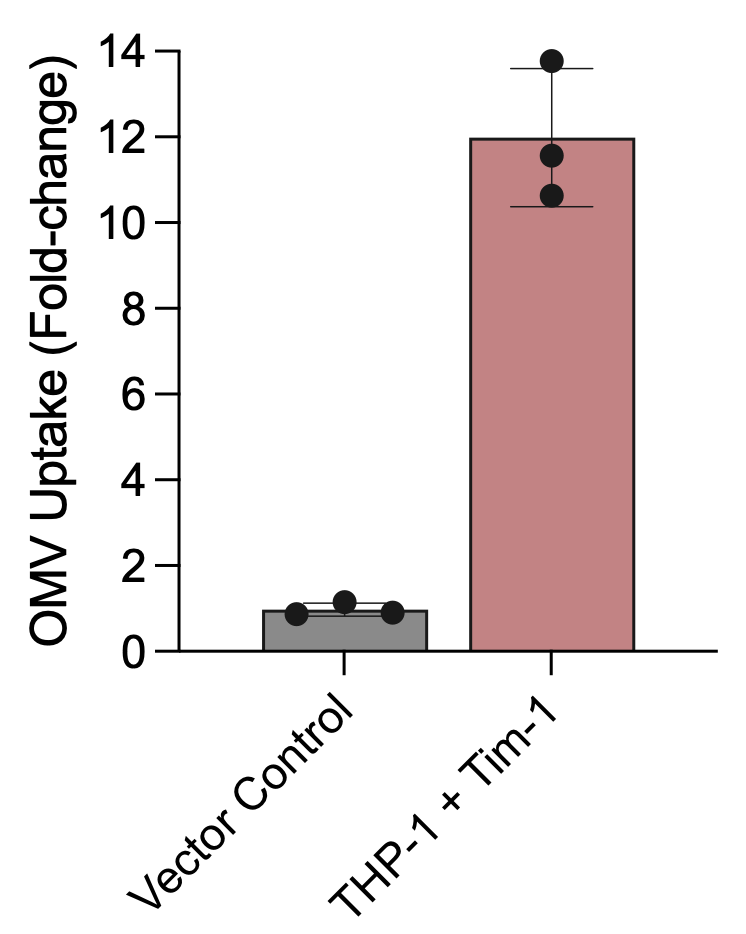


**Fig E. Overexpression of TIM-1 in THP-1 monocytes.** Undifferentiated THP-1 monocytes are treated with 50 µg/mL of DiO stained *E. coli* OMVs for 2 hours and fluorescence (488) determined with flow cytometry. Data is normalized to wildtype THP-1 monocytes.
