# Supplementary material for: Outer membrane vesicles hijack TIM-1 for cellular uptake
Source: PLoS Pathog. 2026 May 26;22(5):e1014256. doi: 10.1371/journal.ppat.1014256 (PMC13225658; doi:10.1371/journal.ppat.1014256)
Supplement: Attachment — Submitted filename: Response_to_Reviewers_PPATHOGENS-D-26-00125R1.pdf [file ppat.1014256.s003.pdf]

Response to Reviewers

Reviewer #1: 1- The role of TIM-1 is investigated exclusively in transformed cell lines, which may represent a phenotype-related bias. Validation of the role of TIM-1, using at least blocking antibody, in non-transformed cell lines (such as HUVEC, IEC-6 etc...) is essential.

We thank the reviewer for their suggestion and have validated the role of TIM-1 in Renal Proximal Tubule Epithelial Cells (RPTEC). This primary cell line is known to express TIM-1 and demonstrated a significant reduction in OMV uptake in the presence of the TIM-1 blocking antibody. This data has been incorporated into Supplemental Figure 1. We have also added discussion regarding the increased expression of TIM-1 observed during injury and the potential role that may play in OMV uptake in vivo (Lines 396 to 401).

2- Using genetically engineered E. coli, the authors have investigated the role of LPS modifications to TIM-1 binding using biolayer interferometry assays. However, uptake of OMVs produced by such E. coli strains (∆wacc, WbbL, mcr-1) have to be evaluated in cellulo, at least in A549 and Caco-2 cells.

We appreciate the reviewers' suggestion and tested OMVs produced by *E. coli* (∆*wacc*, *WbbL*, *mcr-1*) for their ability to be taken up by A549 and Caco-2 cells (Supplemental Figure 4, Lines 228 to 231). All vesicles demonstrated an ability to be internalized by both cell types. OMVs from ∆*wacc* and *WbbL* strains showed an increase in uptake, and OMVs isolated from *mcr-1* expressing *E. coli* reduced uptake.

3- The fact that EHEC's OMV uptake is not impaired in TIM-1 knockout cell line suggests the existence of alternative TIM-1-independent entry routes. This raises the question of how representative OMVs from the model strain E. coli BW25113 are. To exclude a E. coli strain-specific effect, the authors should assess whether TIM-1 - mediated uptake is conserved across additional laboratory, K12, wild-type, pathogenic, or clinical E. coli strains.

We thank the reviewers for their comments and have expanded our testing of TIM-1-mediated uptake with OMVs from additional bacterial species, including the clinical *E. coli* Adherent-Invasive-Escherichia coli (AIEC) strain LF82 as well as *Salmonella* Typhimurium ATCC 14028. Both demonstrated similar TIM-1 dependence to the model *E. coli* BW25113 strain, in which TIM-1 overexpression increases uptake while TIM-1 knockout reduces uptake (Figure 5h, i). We have also discussed the possibility of alternative or redundant receptors in the manuscript (Lines 297 to 299).

Reviewer #2:

1. For the cellular uptake studies, the authors labelled OMVs with a lipophilic dye for flow cytometry analysis. The authors should briefly discuss potential limitations of lipophilic dyes in the Discussion section to better contextualize the uptake process.

We appreciate the suggestion from the reviewer and have discussed the potential limitations of lipophilic dyes such as DiO in the discussion section (Lines 317 to 323).

2. Mention the rationale for choosing the time point (2 hours) for the uptake study.

An incubation time of 2 hours was selected as it provided a strong window between OMV-treated and untreated cells with >95% of cells positive for OMV uptake, and no saturation in signal was observed at this time point. Additional clarity for the 2-hour time point has been provided within the manuscript (Lines 671 to 672).

3. As the authors have already established luciferase expression in E. coli, they should consider performing cellular uptake studies using luciferase-containing OMVs to evaluate OMV internalization. In particular, measuring luminescence would provide an complementary approach to assess TIM-1-mediated OMV uptake.

We appreciate the suggestion by the reviewers and have pursued efforts to quantify OMV uptake using luciferase-containing OMVs. Unfortunately, we were unable to reliably develop an assay capable of measuring differences in OMV internalization even when using chemical blockers of endocytosis such as dynasore. We are currently working to overcome these technical challenges and believe a better approach will be to use a split luciferase system. However, this work is still ongoing and we feel it is beyond the scope of this manuscript.

4. The purity of the OMVs should be carefully assessed. Since only ultracentrifugation (UC) was used for isolation, which may result in crude OMVs mixed with bacterial remnants or medium components, additional purification steps, such as size-exclusion chromatography (SEC) or density gradient centrifugation, are recommended, particularly when receptor-binding interactions are being investigated. This limitation should be briefly acknowledged in the Discussion

We appreciate the comment by the reviewer and clarified that all OMV preps are filtered through a 0.45 μm filter and purified with a 100 kDa size exclusion filter (Lines 655 to 656). We have also acknowledged this limitation in the discussion section (Lines 317 to 323).

5. The authors suggest blocking TIM-1 using PMBN due to its LPS-binding activity. However, since PMBN binds phosphate groups, it is unclear how this might affect TIM-1 interaction with PS. Possible conformation changes? Should discuss the potential impact of PMBN on TIM-1 binding to PS.

We apologize to the reviewer for being unclear about the role PMBN may play in TIM-1 binding LPS and have explained this in detail in the Discussion section (Lines 335 to 343).

6. For studies involving the uptake of OMVs from different bacterial strains (5h- P. aeruginosa; 5i- P. aeruginosa and A. baumannii), the authors should include at least 3 biological replicates for reproducibility.

Thank you for pointing out the limited replicates for these bacterial strains. We have now included data of at least three biological replicates for *P. aeruginosa* and *A. baumannii* in Figure 5h,i.

7. The authors may consider modifying the title of the manuscript, as the data indicate that TIM-1-mediated OMV uptake is strain specific. For example, no uptake of A. baumannii OMVs was observed, and only a ~2-fold increase was seen for E. coli OMVs in TIM-1-overexpressing A549 cells, while only no reduction in uptake for TIM-1 knockout cells.

While we appreciate the suggestion by the reviewer and acknowledge the limitation of not observing TIM-1 dependent uptake for *A. baumannii* OMVs, we believe with the observed dependence of TIM-1 for *F. nucleatum*, EHEC, *P. aeruginosa* as well as recent additional data for AIEC, and *S.* Typhimurium OMVs (Fig. 5h,i) that the title is appropriate.

8. The in vitro results demonstrate that TIM-1 serves as a receptor for E. coli K-12 OMV uptake; however, in vivo systems are more complex. To further evaluate the potential of TIM-1-mediated OMV uptake, the authors should discuss the possibility for a biodistribution study of OMVs in the presence of TIM-1-blocker.

We have added additional discussion regarding the expression of TIM-1 in vivo and the potential relationship between LPS-mediated cell damage and TIM-1 upregulation that could stimulate OMV internalization (Lines 396 to 401). Additionally, we have discussed the importance of future studies looking at OMV distribution in the presence of a TIM-1 blocker (Lines 406-408).

Reviewer #3: Overall, the manuscript is well written and the data are support the conclusions. I have several comments that, if addressed, would further strengthen the study:

1. Additional detail regarding the screened receptor library would be helpful. How was the threshold for defining “significant binders” determined?

We thank the reviewer for their suggestion and have expanded our description of the receptor library within the text (Lines 755 to 760) and described the threshold selection (Line 768).

2. Only TIM-1 (and possibly TIM-4) were validated functionally in cells, whereas other hits from the screen did not alter OMV uptake upon overexpression. Are these additional candidates expressed at the cell surface in the tested epithelial cell lines? If not, this should be stated clearly.

We apologize for the lack of clarity regarding cell line validation. Cell lines in which surface expression was validated are now listed in the Supplementary Table, which includes those used in Fig 2d, Fig 4d, and Fig 5a,b. Notably, we were unable to stain for WBP1 and did not surface

confirm the Caco-2 overexpression strains. We have clarified in the manuscript which strains were and were not confirmed for cell surface expression (Supplemental Table 4, Lines 705 to 710).

3. Relatedly, surface expression of TIM-1 was assessed by flow cytometry, but the corresponding data are not clearly shown – the FACS data in the SI is not easy to interpret in terms of % surface expressed. Similar surface expression validation should be provided for other tested receptors.

We agree with the reviewers' suggestion and have replaced this data with Supplemental Table 4, which now lists all strains surface-validated showing an increase in MFI as well as increases in the % surface expressed. All strains tested demonstrated the expected increases.

4. OMVs were normalized by protein concentration (e.g., 50 μg/mL), but protein content can vary substantially depending on strain background and growth conditions. It would be helpful to provide particle number estimates (e.g., via nanoparticle tracking analysis) and, if possible, an approximate multiplicity of vesicles per cell. Relating the in vitro exposure levels to physiologically relevant concentrations encountered during infection would be helpful.

We agree that protein concentration is not an ideal method to quantify OMVs. We have conducted nanoparticle tracking on our vesicles. We can now estimate the number of particles used in our assays which is included in the manuscript (Line 670). Uptake assays using a concentration of 50ug/mL exposed cells to $6x10^{10}$ particles with particles per cell of ~$6x10^{5}$. While we are hesitant to speculate on the similarity between these concentrations and physiologically relevant concentrations encountered during infection. OMV counts in CF sputum have been observed at ~10^{9} particles/mL (https://doi.org/10.3389/fcimb.2026.1695102). We believe our concentrations are within the range of what is biologically possible, however, much more research is needed to accurately determine OMV concentrations during infection.

5. Please provide more detail on the LPS used in competition assays (Fig. 3). Was it derived from a strain expressing O-antigen or from a rough mutant lacking O-antigen? Because O-antigen is part of LPS, the wording in the manuscript describing “LPS and O-antigen” as separate surface components should also be revised for accuracy.

We thank the reviewer for their suggestions. We have clarified the LPS used in the BLI assays as LPS-EK (Invitrogen), which is from the rough LPS strain *E. coli* K12 (Lines 748 to 749).

6. The TIM-1 overexpression and knockout data are convincing, but additional context would strengthen the physiological relevance. Can the authors comment on endogenous TIM-1 expression levels in the tested epithelial cell lines and how they compare to primary epithelial or immune cells in vivo? Since TIM-1 expression is often inducible

under stress or injury conditions, this discussion would help frame the in vivo implications.

We thank the reviewer for their suggestion and have validated the role of TIM-1 in Renal Proximal Tubule Epithelial Cells (RPTEC). This primary cell line is known to express TIM-1 and demonstrated a significant reduction in OMV uptake in the presence of the TIM-1 blocking antibody. This data has been incorporated into Supplemental Figure 1. We have also added discussion regarding the increased expression of TIM-1 observed during injury and the potential role that may play in OMV uptake in vivo (Lines 396 to 401).

7. The experiment expressing TIM-1 in monocytes to assess cell death requires clearer justification. What is the relevance? Monocytes are known to internalize OMVs and mount inflammatory responses in the absence of TIM-1.

We apologize for the confusion regarding the use of monocytes. They were used due to their known susceptibility to OMV-induced inflammatory response. We were interested in testing if TIM-1-dependent uptake would result in increased downstream inflammation. We have incorporated this additional clarity into the manuscript (Lines 276 to 277).

Part III – Minor Issues: Editorial and Data Presentation Modifications
Please use this section for editorial suggestions as well as relatively minor modifications of existing data that would enhance clarity.

Reviewer #1: While the authors underline the need to identify additional receptors, involvement of receptor-independent uptake mechanisms, such as direct membrane fusion, have been described and merit discussion.

We agree with the reviewer and now discuss membrane fusion as an OMV uptake mechanism (Lines 79 to 81).

The manuscript would benefit from a more comprehensive discussion of other putative OMV receptors including pattern recognition receptors (PRRs), particularly Toll-like receptors that detect M/PAMPs such as LPS, flagellin, and peptidoglycan, as well as membrane glycolipids such as ganglioside GM1 (the cholera toxin receptor) or globotriaosylceramide Gb3 (the Shiga toxin receptor)...

We have added a more comprehensive discussion on other putative OMV receptors (Lines 86 to 88).

The first paragraph of the discussion resumes arguments already presented and extensively developed in the introduction and should be shortened or removed.

We have significantly shortened the length of the first paragraph in the discussion section.

Reviewer #2: (No Response)

Reviewer #3: 8. The wording describing the OMV surface composition should be revised: O-antigen is part of LPS and should not be described as a separate surface component.

We have corrected the language when describing O-antigen throughout the manuscript.

9. The pink and purple colors used in some graphs are difficult to distinguish.

We have replaced the pink/purple colors used to improve readability.

10. Fonts in the Supplementary Information are inconsistent and should be standardized.

Fonts have been corrected throughout the Supplemental Information.
